# Thermosonication enhanced the bioactive, antioxidant, and flavor attributes of freshly squeezed tomato juice

**DOI:** 10.1016/j.ultsonch.2025.107299

**Published:** 2025-03-02

**Authors:** Limei Li, Hang Su, Lingling Pang, Yanfang Pan, Xihong Li, Qing Xu, Jitian Song, Liping Qiao

**Affiliations:** a Tianjin Key Laboratory of Integrated Design and On-line Monitoring for Light Industry & Food Machinery and Equipment, College of Mechanical Engineering, State Key Laboratory of Food Nutrition and Safety, College of Food Science and Engineering, Tianjin University of Science and Technology, Tianjin 300457, China; b Institute of Western Agriculture, Chinese Academy of Agricultural Sciences, Changji 831100, China; c Institute of Food Science and Technology, Chinese Academy of Agricultural Sciences, Beijing 100193, China; d Tianjin Gasin-DH Preservation Technology Co., Ltd., Tianjin 300300, China

**Keywords:** Sterilization, Thermosonication, Bioactive components, Freshly squeezed tomato juice

## Abstract

The sterilization of freshly squeezed juices typically results in a compromise to the natural flavor, bio-active components, and nutritional value. Developing a new sterilization method for controlling the diverse effects has emerged as a significant challenge. This research aims to explore the potential application of thermosonication (TS) for freshly squeezed tomato juice. Results revealed that both TS (temperature 50, 60, and 70 ℃; time 5, 10, and 15 min) and thermal pasteurization (TP, 85 ℃/10 min) effectively inactivated microorganisms. No significant differences were observed in the basis properties (pH, total soluble solids (TSS) and titratable acidity (TA)) of tomato juice. Notably, TS effectively enhanced the juice quality, and the optimal condition is TS 60 ℃ 15 min. Its retention rates in color and suspension stability were greatly enhanced. Meanwhile, TS (60 ℃, 15 min) not only significantly increased lycopene content (42.13 %), ascorbic acid content (36.64 %), flavonoids content (33.94 %), and total phenols content (34.06 %), but also maintain a higher antioxidant capacity compared to PJ samples. Moreover, the sensory quality and volatile substances of TS treated were enhanced. It can be inferred that TS through ultrasonic cavitation ensured the microbial safety, improved nutritional value and sensory quality of tomato juice. This report provide a basis that a proper lower pasteurization temperature produced better effect in tomato juice when combined with ultrasound.

## 1 Introduction

Sterilization constitutes an indispensable process in the production of freshly squeezed juices, as it exerts a considerable influence on the nutritional value, storage characteristics and shelf-life of the products[1]. However, the traditional sterilization techniques for fresh juice are mainly thermal sterilization, including thermal pasteurization (TP), high temperature short-term sterilization (HTST) and ultra-high temperature instantaneous sterilization(UHT), which is easy to cause the degeneration in sensory attributes, the destruction in heat-sensitive nutrients and the decline in suspension stability[1]. Developing a new sterilization method for controlling the diverse effects is to be significant challenge and of great importance.

Ultrasound, as a highly efficient, environmentally friendly, and energy-saving non-thermal assisted sterilization technology, has currently emerged as a focal point of research in food processing. In contrast to the mechanisms of high-temperature or long-term sterilization, the collapse of cavitation micro-bubbles generate mechanical shear force (1.5 kg/cm^2^) and local instantaneous high temperature & pressure (5000 K, 1800 atm)[2]. The sonoporation phenomenon, induced by ultrasonic cavitation, plays a critical role in the process of microbial inactivation. It cause the disruption of cell membrane integrity, an increase in membrane permeability, and the leakage or denaturation of certain intracellular functional substances, including enzymes and DNA, ultimately resulting in irreversible cell death[2], [3]. Meanwhile, the chemical effect of ultrasonic cavitation generates free radicals (OH^–^, H_2_O_2_), which can boost oxidative stress reaction, interfere the energy metabolism, and ultimately induce microorganisms apoptosis[4]. Considering its synergistic effect and limitations, the application of ultrasound combined with proper lower pasteurization temperature may produce equivalent sterilization effect or potentiate its action. The collapse of ultrasonic cavitation bubbles can trigger strong shock waves and microscopic turbulence[5]. The synergy of ultrasound and heat can enhance fluid convection heat transfer, improve heat transfer efficiency, accelerate the inactivation of intracellular enzymes and microbial death, thereby achieving a synergistic sterilization effect[6]. Additionally, ultrasonic cavitation has a homogenizing effect, which can enhance the suspension stability of fruit juices[7], and promote the liberation of bioactive components from cellular tissue complexes to the liquid phase[8]. Its super effects was also reported in posotia juice[9], inulin prebiotic beverage[10] and strawberry juice[11] in maintaining the stability, color, aroma, bioactive ingredients, and antioxidant activities.

The tomato (*Solanum lycopersicum* L.), a nearly spherical and bright red berry, is one of the foremost extensively cultured and consumed fruits and vegetables globally. Particularly, the tomato is naturally rich in vitamin C, carotenoids, lycopene, flavonoids, polyphenols and other bioactive substances, which have antioxidant, antitumor, and cardiovascular protective effects[12]. Besides hydrodynamic cavitation[13], high pressure homogenization[14], and UV-LED technology[15], there are few studies on the sterilization technology of tomato juice. This research aims to find out the effects of TS parameters on microbial inactivation, bioactive compound, and sensory attributes. Hoping to develop a theoretically based method for the prospective sterilization in tomato juice production.

## 2 Materials and methods

### 2.1 Material and tomato juice samples preparation

The ‘Jinguan No.5′ tomatoes used in this study were mature and fresh, with a uniform red surface color (L* = 38.56, a* = 17.34, b* = 9.41) and an average single fruit weight of 480–560 g. These tomatoes were bought from a local market. Tomatoes were washed with water and cut into chunks. Then, these tomato chunks and distilled water with the ratio of 1:2 were put into an electric juice extractor (MJ-PB12, Media, Guiyang, China) and juiced for 2 min. Fresh-squeezed tomato juices was removed large particles and impurities by filtering with 4 layers of sterile muslin gauze (HYNAUT, Qingdao, China).

### 2.2 Thermal pasteurization (TP) and thermosonication (TS) treatment

For TP treatment, 120 mL of tomato juice was transferred into a sterilized erlenmeyer flask and pasteurized at 85 ℃ for 10 min in an electron-thermostatic water bath (DK-98–1, Taisite, Tianjin, China). For TS treatments, 120 mL of tomato juice was filled in sterilized erlenmeyer flask and placed in an ultrasonic bath (SB-4200 DTD, Xinzhi, Ningbo, China) operated at an amplitude of 99 % (480 W). The TS parameters investigated in this study included three temperatures (50 ℃, 60 ℃, and 70 ℃) and three durations (5 min, 10 min, and 15 min). The treatments were accordingly designated as TS 50–5, TS 50–10, TS 50–15, TS 60–5, TS 60–10, TS 60–15, TS 70–5, TS 70–10, and TS 70–15. The tomato juice samples of different treatments was named as follows: freshly squeezed (untreated) tomato juice (FJ), TP treated tomato juice (PJ), and TS treated tomato juice (TJ). After treatment, these samples were immediately cooled in a water bat and subsequently stored at 4 ℃ and −80 ℃ (902-ULTS, Thermo Fisher Scientific, US) apart for further analysis.

### 2.3 Microbiological analysis

The total bacteria(TB), yeast and mold (Y&M) and *Escherichia coli* (*E. coli*) were detected based on the approach of Wang et al.[16] with minor adjustments. 1 mL of tomato juice sample was diluted up to 10^-1^ using 9 mL of distilled water, in turn, until the sample was diluted to 10^-4^. Subsequently, 0.1 mL of diluted sample was coated on the Luria-Bertani (LB, TB), potato glucose (PDA, Y&M) and Violet Red Bile Agar with Lactose (VRBA, *E. coli*) plates, respectively. After that, the LB and VRBA plates were placed and incubated at 37 ℃ for 48 h, while the PDA plates at 30 ℃ for 5 d. The number of microorganisms were presented as log CFU/mL.

### 2.4 Color and browning index (BI) analysis

The color parameters of L*, a*, b*_,_ and ΔE were evaluated in light of the approach of Wu et al.[17] using a colorimeter (WR-18, Shenzhen, China). ΔE = [(L* − L_0_)^2^ + (a* − a_0_)^2^ + (b* − b_0_)^2^]^1/2^.

The BI was estimated as Das et al. [9] described with slight modifications. 5 mL of 95 % ethanol was blended with equal volume of tomato juice samples. Then, the mixed juice samples were centrifuged at 4 ℃, 6000 × *g* for 15 min in a centrifuge (TGL-16, Shu Ke, Chengdu, China). Then, the supernatant was collected and determined at the absorbance of 420 nm by a microplate reader (SpectraMax190, MEGU, Shanghai, China). The 80 % ethanol solution was used for control group. The BI was computed as follows. BI = A × V_1_/V_2_, where, A represented the absorbance value, V_1_ indicated the volume of extraction liquid (mL), V_2_ signified the volume of juice sample.

### 2.5 pH, total soluble solids (TSS), titratable acidity (TA), and TSS/TA analysis

The pH value was assessed employing one digital pH meter (PHS − 3E, Shanghai, China). The TSS was estimated by means of one handheld digital refractometer (PAL-3, Japan) and represented in °Brix. TA was determined using titration on the basis of Alam et al.[18].

### 2.6 Sedimentation index (SI) and cloudiness (CL) analysis

The SI was estimated in line with the approach proposed by Giordano et al.[19]. 10 mL of tomato juice samples were poured into a graduated centrifuge tube. Then, these tested tubes were arranged in a rack and kept at 25 ℃ for 7 d. The SI was calculated using the following equation. SI (%) = V_s_/V_t_ × 100, where, V_s_ and V_t_ represented the sedimentation volume and the total volume, respectively. The CL was estimated based on the approach of Das et al.[9] with minor adaptations. 5 mL of tomato juice was centrifuged by one centrifuge (TGL-16, Shu Ke, Chengdu, China) at 4 ℃, 6000 × *g* for 20 min. The supernatant was then taken and gauged by a microplate reader (MD SpectraMax190, MEGU, Shanghai, China) at the absorbance of 660 nm, with distilled water as the control group. The CL was computed as follows. CL (%) = (C_a_/C_o_) × 100, where, C_a_ and C_o_ represented the absorbance of the tomato juice and control group, respectively.

### 2.7 Antioxidant contents analysis

#### 2.7.1 Total phenolic content (TPC) analysis

The TPC of tomato juice was estimated in line with the approach detailed by of Kalsi et al.[20] with with certain adjustments. 2 mL of tomato juice sample was diluted with 30 mL distilled water. Then diluted juices was blended with 2.5 mL of 10 % Folin–Ciocalteu phenol solution. Following a reaction of 1 min, 7.5 mL of 20 % Na_2_CO_3_ was solution was introduced into the mixture. The resulting solution was then adjusted to a final volume of 50 mL using distilled water and incubated at 75 ℃ for 10 min. Thereafter, an aliquot of 300 μL from this mixture was taken for absorbance measurement at 760 nm utilizing a microplate reader (SpectraMax190, MEGU, Shanghai, China). Results were noted as μg/mL equivalent to gallic acid (μg GAE/mL). The calibration curve employed with gallic acid was as follows: y = 0.1185x + 0.0136, R^2^ = 0.9967.

#### 2.7.2 Total flavonoids content (TFC) analysis

The TFC of tomato juice was estimated based on the approach of Kalsi et al.[20] with specific adjustments. 0.5 mL of the tomato juice was blended with 0.45 mL in 1:2 vol ratio between sodium nitrite (5 %) and aluminum chloride (10 %) solution and reacted for 5 min. Then, the mixture was mixed with 1 mL of sodium hydroxide solution (1 mol/L) and adjusted the final volume to 5 mL using distilled water. Thereafter, 300 μL of the final mixture was taken and the absorbance was surveyed using a microplate reader (SpectraMax190, MEGU, Shanghai, China) at 510 nm. Results were noted as μg/mL equivalent to catechin (μg CE/mL).

#### 2.7.3 Ascorbic acid content (AAC) analysis

The AAC of tomato juices was assessed based on the approach of 2,6-Dichlorophenol-Indophenol dye (DID) titration[21]. 10 mL of tomato juice was mixed with 90 mL 20 g/L oxalic acid solution. After 10 min, the mixture was filtered. 10 mL of the filtrated mixture was titrated using standardized DID until the filtrate exhibited a lighter pink colouration and no fading within 15 s. 10 mL 20 g/L oxalic acid solution was utilized as the control. Results were indicated as μg/mL.

#### 2.7.4 Total lycopene content (TLC) analysis

The TLC of the tomato juice samples was conducted in accordance with the approach of Dai et al.[22]. 2 mL of tomato juice sample was added into 25 mL extraction solvents of hexane, ethanol, and acetone (2:1:1, v/v/v). Then the juice sample was extracted for 20 min at 25 ℃ in the dark with a vortex. Following centrifugation at 4 ℃, 6000 × *g* for 10 min (TGL-16, Shuke, Sichuan, China), the supernatant was collected for following measurement. Subsequently, the residue was extracted a further 2 times. These collected supernatants were then blended and used as the extracting solution. The absorbance of extracting solution was read at 503 nm. The hexane was employed as the control. TLC (μg/mL) was calculated using the following the equation. TLC (μg/mL) = A_503_ × M_r_ × n × 1000 / L × ε, where, ε indicated the extinction coefficient of lycopene (172000 L/mol∙cm), M_r_ denoted the molecular weight of lycopene (536.9 g/mol), L denoted the optical path (1 cm), n represented dilution ratio.

### 2.8 Antioxidant capacity analysis

#### 2.8.1 2,2-Diphenyl-1-picrylhydrazyl (DPPH) radical scavenging capacity analysis

DPPH free radical scavenging capacity was evaluated on the basis of Shen et al.[23]. 2 mL of 0.2 mM ethanolic DPPH solution (75 % ethanol) was added into 2 mL of tomato juice sample and then incubated at 25 ℃ in the dark for 30 min. After that, the mixture was taken and centrifuged using one centrifuge (TGL-16, Shu Ke, Chengdu, China) at 4 ℃, 6000 × *g* for 15 min. The absorbance of supernatant was measured using a microplate reader (MD SpectraMax190, MEGU, Shanghai, China) at 517 nm. Results were computed as follows: DPPH activity (%) = (A − A_0_)/A × 100, whereby, A_0_ and A represented the absorbance of distilled water and tomato juice sample, respectively.

#### 2.8.2 ABTS radical scavenging ability analysis

ABTS radical scavenging ability was determined as described of Ramírez-Melo et al.[24]. The equal volume of ABTS (7 mM) and K_2_S_2_O_8_ (2.45 mM) was mixed and subsequently incubated at 25 ℃ in the darkness for 12 h. Then, the prepared ABTS free-radical stock solution (ABTS^+^) was successively diluted 20–30 times using 100 % ethanol until the absorbance value reached 0.700 ± 0.002 at 734 nm. 0.4 mL of tomato juice sample was added to 3.6 mL of the ABTS working solution (diluted ABTS^+^) and incubated for 10 min in the dark. 300 µL of treated sample was subsequently aspirated into a 96-well plate and read at the absorbance of 734 nm, with the distilled water replaced the tomato juice sample as a control. ABTS activity (%) = (A_0_ − A)/A_0_ × 100, whereby, A_0_ and A denoted the absorbance of distilled water and tomato juice, respectively.

### 2.9 Electronic nose analysis

The flavor of tomato juice samples were analyzed on the basis of Xu et al.[11]. 10 mL of tomato juice was poured into a headspace vial and incubated in a water bath at 40 ℃ for 20 min. Then, flavor changes were detected using an electronic nose system.

### 2.10 Sensory evaluation

The sensory evaluation of tomato juice samples was performed in line with the approach of Bao et al.[25]. The evaluation panel consists of 15 males and 15 females, aged between 20 and 35 years. All members had received systematic training in food sensory evaluation. Meanwhile, The tomato juice samples were randomly numbered and provided to each evaluator, who was required to score the sensory parameters of color, aroma, taste, appearance and total acceptability.

### 2.11 Statistical analysis

Each treatment was tested in triplicate. Results were presented as mean ± standard deviation (SD). The analysis of the data utilized Origin 2018 software (Microcal Software, MA) and SPSS 27 software (SPSS Inc., USA). A one-way analysis of variance (ANOVA) was performed using the Duncan multiple comparison post hoc test, and the level of significance was set at *p* ＜ 0.05.

## 3 Results and Discussion

### 3.1 Microbiological analysis

As summarized in Table 1, the TS treatments produced a significant reduction in the levels of microorganisms of tomato juice samples, as opposed to the untreated juice (*p* < 0.05). As TS temperature and duration increased, the reduction of microorganisms exhibited a logarithmic decline. Following TS treatment at 50 ℃, the number of TB, Y&M and *E.coli* in tomato juice were found to be 3.37–2.13 log CFU/mL, 2.36–1.03 log CFU/mL, and 2.40–1.18 log CFU/mL, respectively. The observed decline in microorganisms an be primarily attributed to a combination of chemical and mechanical impacts induced by ultrasound, including the ultrasonic cavitation, generation of free radicals (H^+^ and OH^–^), and mechanical forces (shear forces, shock waves), which led to the cellular damages and eventual inactivation[26]. Notably, The microorganisms of TB, Y&M and *E.coli* were not completely inactivated at TS 50 ℃. Similarly, it has been demonstrated that these microorganisms were incapable of being inactivated eventually when subjected to thermosonication temperatures below 50 ℃[18]. The TB, Y&M, and *E.coli* were not detectable through thermosonication at 60 ℃ for 15 min, which achieved an equivalent effect to that of pasteurization at 85 ℃ for 10 min. Our results confirmed that the synergistic effect of mild heat and low-frequency ultrasound could result in a complete inactivation of microorganisms[27].

**Table 1:** Effects of different sterilization methods on the number of microorganisms in tomato juice.

Treatments	TB Count (log CFU/mL)	Y& M Count (log CFU/mL)	E.coli Count (log CFU/mL)
FJ	3.81 ± 0.04^a^	2.73 ± 0.02^a^	2.29 ± 0.02^a^
PJ	ND^e^	ND^e^	ND^d^
TS50-5	3.37 ± 0.01^b^	2.36 ± 0.02^b^	2.40 ± 0.10^b^
TS50-10	2.81 ± 0.02^bc^	1.93 ± 0.01^c^	1.85 ± 0.03^bc^
TS50-15	2.13 ± 0.04^c^	1.23 ± 0.01^d^	1.18 ± 0.03^c^
TS60-5	1.87 ± 0.02^c^	1.07 ± 0.03^cd^	1.14 ± 0.01^c^
TS60-10	1.05 ± 0.01^d^	ND^e^	ND^d^
TS60-15	ND^e^	ND^e^	ND^d^
TS70-5	ND^e^	ND^e^	ND^d^
TS70-10	ND^e^	ND^e^	ND^d^
TS70-15	ND^e^	ND^e^	ND^d^

Note: Data were expressed as Means ± SD (n = 3). Values noted identical superscript letters in the same column indicated no statistically significant difference. The values that differ in superscript lettering presented significant (*p* < 0.05). ND represented no detectable microorganisms.

### 3.2 Appearance and color stability analysis

**Fig. 1:**
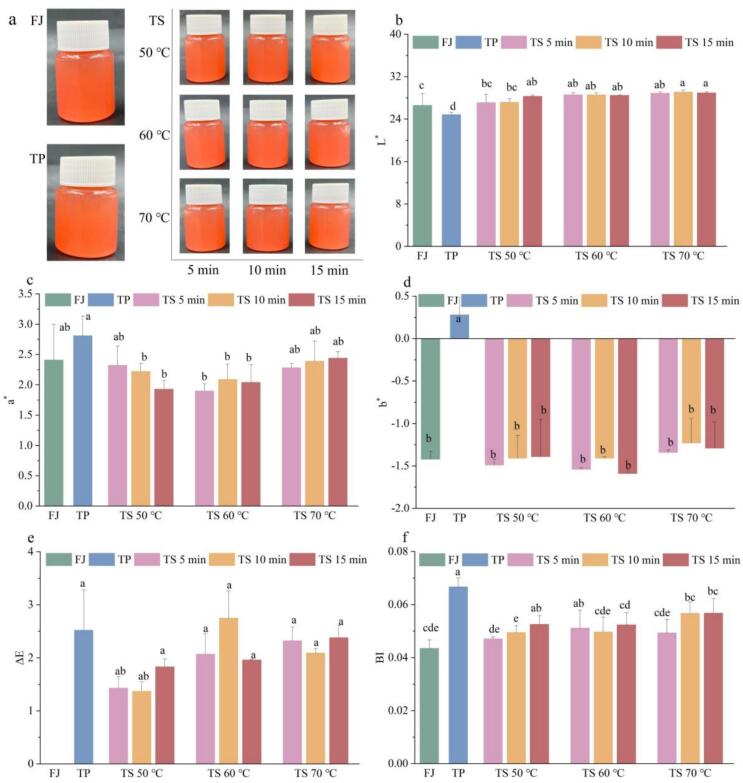
The appearance (a), color attributes (L* (b), a* (c), b* (d), ΔE (e)), and BI (f) of tomato juice samples sterilized using different methods. Different letters presented above the error line indicate significant differences between the samples (*p* ＜ 0.05).

There were no discernible visual differences in the color of tomato juice following various sterilization methods (Fig. 1a). The L* values (Fig. 1b) and b* values (Fig. 1d) for the samples treated with TS showed an increase, whereas the a* values (Fig. 1c) exhibited a decrease in comparison to the FJ and PJ samples. Specifically, when juxtaposed with the FJ (26.58 ± 2.21, 2.41 ± 0.59, −1.42 ± 0.09) and PJ (24.83 ± 0.44, 2.81 ± 0.32, 0.28 ± 0.83) samples, the L* values (27.10 ± 1.61 ∼ 29.38 ± 0.39) of the TS treated juices showed a significant rise (*p* < 0.05), while not all a* and b* values were significantly altered. It is suggested that ΔE values exceeding 3.0 could indicate a notable visual difference. The ΔE (1.37 ∼ 2.38) (Fig. 1e) revealed no significant differences among all treatments (*p* > 0.05). The observed rise of L* may be ascribed to the shear force exerted by ultrasound, which fragmented larger protein fractions and cellular tissues in tomato juice into smaller particles, leading to increased uniformity and improved light transmittance[14]. Similar phenomenons have also been observed in peach juice treated with ultrasound technology[28]. The reductions in a* values along with the boosts in b* values might be associated with the cavitation effects of ultrasound. It can be deduced that carotene, lycopene, and chlorophyll were liberated from the compromised cell membranes, where they could potentially undergo oxidation by radicals[14]. Nevertheless, after TS treatments at 70 ℃ for 10 and 15 min, both a* and b* values rose, likely due to the elevated temperature intensified pigment degradation and enzymatic browning[23].

The effects of differing sterilization methods on BI values of tomato juice were represented in Fig. 1f. The BI for the FJ and PJ samples were 0.044 ± 0.003 and 0.067 ± 0.003, respectively, whereas the TJ samples displayed a range from 0.047 ± 0.003 to 0.056 ± 0.005. The findings demonstrated that the BI of the PJ sample exhibited a statistically significant difference (*p* < 0.05) from those of FJ and TJ samples. It was also noted that the BI of the TS treated tomato juices presented a minor increase in response to an prolonged thermosonication duration and raised temperatures. This rise in BI may be linked to the Maillard reaction as well as the thermal decomposition of pigments such as carotenoids and chlorophyll. Our findings align with prior research conducted on the posotia juice and lapsi juice[9], [29]. Moreover, It has been suggested that the loss in ascorbic acid may be one of the reasons for the elevated BI values seen in red grape juice[30].

### 3.3 Basic quality analysis

**Fig. 2:**
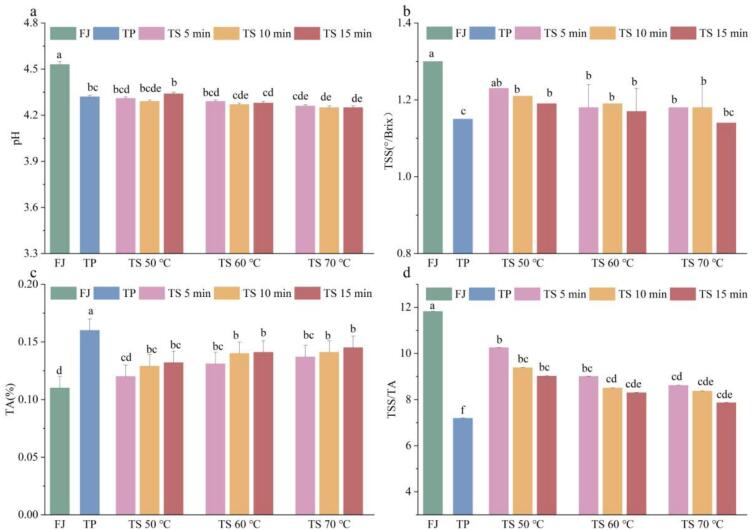
The pH (a), TA (b), TSS (c), and TSS/TA (d) of tomato juice samples sterilized using different methods. Different letters presented above the error line indicate significant differences between the samples (*p* ＜ 0.05).

As illustrated in Fig. 2, the FJ samples exhibited the pH, TSS, and TA values were 4.53 ± 0.02, 1.3 ± 0.06 °Brix, and 0.11 ± 0.01 %, respectively. After implementing TP and TS treatments, both the pH and TSS levels in tomato juice decreased, while there was an increase in TA. Notably, no statistically significant difference was detected between the TS treated tomato juices and the untreated tomato juice. Furthermore, no notable differences were identified among the TS treated samples (*p* ＞ 0.05). Our results corroborated the fact that TS treatment did not alter the basic qualities of melon juice as documented by Liu et al.[31]. The TA content in TS treated juices exhibited a relatively closer value to that of the FJ samples than to the PJ samples. This phenomenon may be linked to the liberation of certain bound organic acids duo to heat and ultrasonic cavitation, leading to the leakage of intracellular H^+^[32]. The decrease in TSS may be associated with the dissolution of fructose caused by Maillard reaction and ultrasonic cavitation[31]. Overall, the tomato juice treated by TS were closer to FJ samples in pH, TSS, and TA, which had a natural flavor without adjusting the sugar acid compound artificially.

### 3.4 Suspension stability analysis

**Fig. 3:**
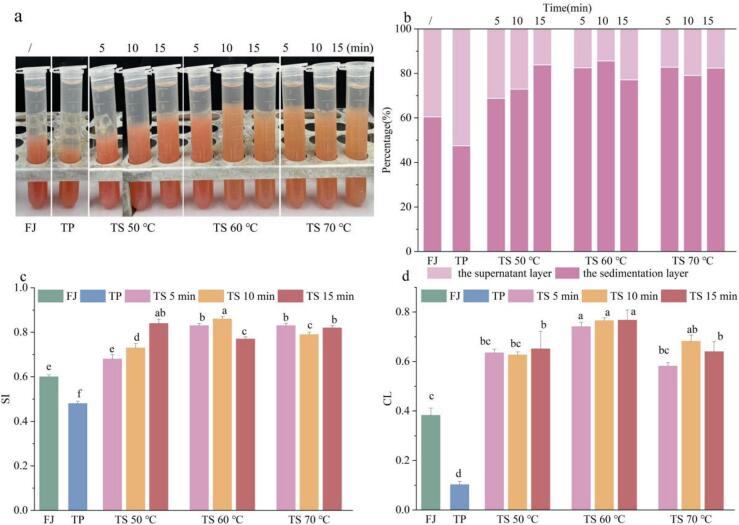
The stratification appearance image (a), stratification percentage (b), SI index (c), and CL (d) of tomato juice samples sterilized using different methods. Different letters presented above the error line indicate significant differences between the samples (*p* ＜ 0.05).

The suspension stability of tomato juices was visually observed and illustrated in Fig. 3a-b. After 7 days’ storage at 25 ℃, both the FJ and PJ samples exhibited significantly severe stratification, with SI values (Fig. 3c) recorded at 0.60 ± 0.01 and 0.48 ± 0.01, respectively. Nevertheless, the TS treated samples demonstrated a significantly enhanced resistance to sedimentation, with the SI values ranged from 0.63 ± 0.02 to 0.86 ± 0.01 (*p* ＜ 0.05). The highest SI value for the tomato juices was observed at TS 60 ℃ for 10 min, reaching a value of 0.86. Furthermore, it was noted that the increasing thermal ultrasonic processing time and temperature led to the elevated SI values. The elevated SI value observed in the TS treated samples can be ascribed to the diminution of particle size resulting from the process of ultrasonic cavitation[28]. The method of preventing agglomeration by ultrasonic cavitation was also proposed in apple juice and peach juice[10], [28]. The tomato juice samples treated at TS 60 ℃ for 10, 15 min, and at TS 70 ℃ showed discoloration during storage for 7d, which was possibly due to the degradation of lycopene. Specifically, all-trans lycopenes underwent irreversible transformation into mono-poly cis forms, which exhibited higher reactivity and lower chromatic properties. Meanwhile, these forms of lycopenes were oxidized, leading to a loss of color and an off-flavor in the tomato juice[33].

As depicted in Fig. 3d, the CL value of the FJ sample was determined to be 0.38 ± 0.03, while that of the PJ sample was 0.11 ± 0.01. Specifically, the CL values of the TS treated samples presented a range of 0.64 ± 0.01 to 0.76 ± 0.04. CL values of tomato juice reached its peak at TS 60 ℃ for 10 min, with a value of 0.76. It was considered that the CL value was intimately associated with components of pectin, protein, cellulose, and hemicellulose[9]. The protein and pectin macro-molecules were fragmented into smaller particle matters by ultrasonic cavitation, which facilitated particle dispersion and phase transparency. Additionally, the synergistic effect of heat and ultrasound led to a greater number of macro-molecules being broken down. Thus, the smaller particles and enhanced dispersion in tomato juices might account for the higher cloudiness values[20]. These results were in line with those of prior studies on thermosonication in guava and blackcurrant juices[20], [34].

### 3.5 Antioxidant contents

**Fig. 4:**
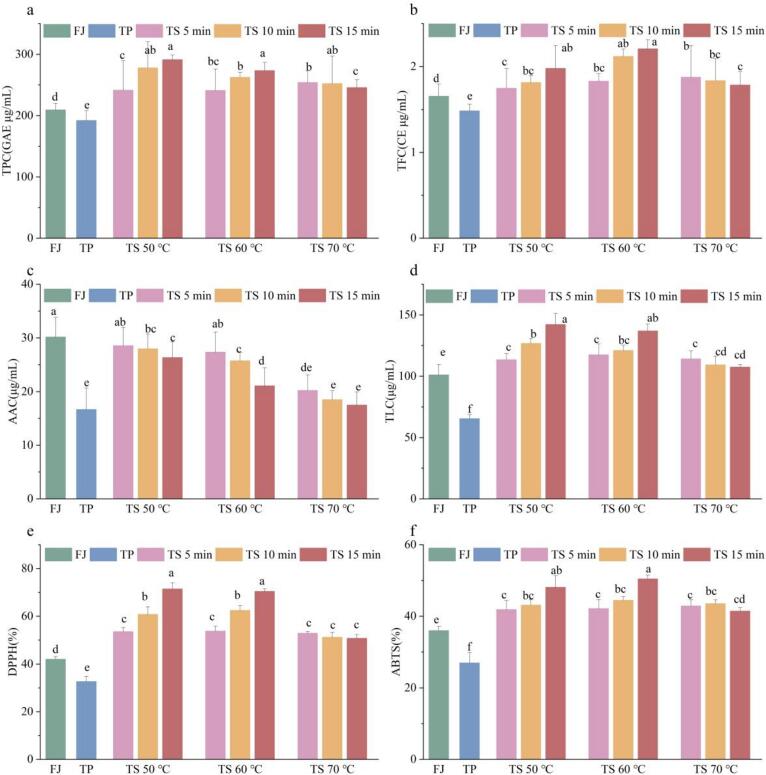
The TPC (a), TFC (b), AAC (c), TLC (d), DPPH (e), and ABTS (f) of tomato juice samples sterilized using different methods. Different letters presented above the error line indicate significant differences between the samples (*p* ＜ 0.05).

The TPC of the FJ and PJ samples were 209.37 ± 10.60 and 192.13 ± 15.97 GAE μg/mL (Fig. 4a), respectively. It was noted that TPC for the TP treated sample was markedly reduced (*p* < 0.05) in comparison to the untreated tomato juice. Conversely, a a notable rise (*p* < 0.05) in TPC was detected among all TS treated tomato juices in comparison to the FJ sample. The TPC was demonstrated to be notably increased (*p* < 0.05) at TS 50 ℃ and TS 60 ℃, with the values ranging from 241.35 ± 42.17 GAE μg/mL to 291.37 ± 7.36 GAE μg/mL. Among these, the maximum value was detected at TS 50 ℃ for 15 min. The observed increase in TPC could potentially be ascribed to the additional joint action of ultrasonic cavitation and heat-induced softening of matrices, resulting in the cell disruption and the liberation of bound phenols[35]. In addition, the chemical effect of cavitation generated OH^–^ radicals, which facilitated the hydroxylation of phenolic aromatic rings, consequently leading to an increase in phenols[36]. It was notable that there was a minor decline in TPC with an increasing duration at TS 70 ℃, which might be ascribed to non-enzymatic browning of total phenols under prolonged exposure to high temperature[37]. Similarly, it has been documented that the TPC was reduced in thermosonicated guava juices at 80 ℃[20].

Similarly, the variation in TFC of tomato juices exhibited a comparable pattern to that of TPC (Fig. 4b). The TFC of the FJ and PJ samples was determined to be 1.65 ± 0.14 CE μg/mL and 1.48 ± 0.07 CE μg/mL, respectively. Conversely, the TFC of TS-treated samples exhibited a significant (*p* < 0.05) rise, with the contents ranging from 1.75 ± 0.23 to 2.21 ± 0.11 CE μg/mL. With regard to the tomato juice samples treated at TS 50 ℃ and TS 60 ℃, the TFC of the tomato juices displayed an increased tendency in response to both the rise in temperature and duration. The possible explanation might be that the continuous action of ultrasound caused the bound phenolics attached to the cell wall and fibrous tissue residues to be free phenolics, resulting in an increase in flavonoids[38]. The maximum value of TFC (2.21 ± 0.11 CE μg/mL) was observed at TS 60 ℃ for 15 min, which was increased by 33.94 % and 49.32 %, respectively, compared to the FJ and PJ samples. Nevertheless, the TFC presented a slight decline with the prolongation of ultrasonic time at TS 70 ℃. This could be attributed to the non-enzymatic browning under the excessive temperature[25].

As depicted in Fig. 4c, the AAC of the PJ sample (16.66 ± 4.01 μg/mL) and TS treated samples (17.50 ± 2.5 ∼ 28.56 ± 3.41 μg/mL) exhibited a significant reduction of 46.79 % and 5.37 % ∼ 42.01 %, respectively (*p* < 0.05), in contrast to the FJ sample (30.18 ± 3.64 μg/mL). As the duration of thermal-ultrasonic treatment increased, the AAC exhibited a gradual decline, which might be attributed to the instantaneous high temperatures generated by cavitation. In addition, the decline in ascorbic acid might be linked to the sonochemical yielded that produced OH^–^ and H^+^ free radicals, along with H_2_O_2_[39]. As the thermal-ultrasonic temperature rose, a corresponding decline in AAC of tomato juices was detected. The above phenomenon may be ascribed to the heating process, which has been demonstrated to lead to a significant degree of decomposition of ascorbic acid[40]. Besides, the cold ultrasound treatment (CUT, 10 ℃) led to a significant preservation in ascorbic acid levels in tomato paste, which primarily benefited from the inhibitory action of low temperature[41].

It was apparent that there was a notable (*p* < 0.05) increment in TLC of the whole TS treated tomato juices compared to the FJ samples (Fig. 4d). A notable increase in TLC was observed after TS treatments at 50 ℃, 60 ℃, and 70 ℃, with values rising by 12.35–42.13 %, 16.38–39.86 %, and 6.36–13.11 %, respectively. The TLC increased gradually with increasing thermal-ultrasonic time and reached the peak value (142.20 ± 9.23 μg/mL) at TS 50 ℃ for 15 min. Prior research has indicated that ultrasound enhanced the lycopene content in a range of juices, including pumpkin, orange, and tomato[41], [42], [43]. The rise in lycopene levels was likely attributable to the mechanical shear forces induced by the cavitation process, which disrupted cellular structure and liberated lycopene molecules from the lycopene-protein complexes into the tomato juice matrix[44]. Another potential explanation might be that the lipoxygenase (LOX) were inactivated by the cavitation, resulting in the lycopene not being oxidized[45]. There was an insignificant (*p* ＞ 0.05) reduction in lycopene with increasing ultrasound time at TS 70 ℃, which may be attributed to the thermal degradation of lycopene occurring[46].

### 3.6 Antioxidant capacity

As depicted in Fig. 4e-f, a significant (*p* ＜ 0.05) difference was manifest in antioxidant activity when comparing the FJ and TP treated samples, with values of 42.05 % and 36.00 %, respectively. The DPPH and ABTS values of TS treated tomato juices rose by 8.79–29.45 % and 5.46–14.42 %, respectively, when compared to the FJ sample. It was previously published that the antioxidant activity of apple beverage also demonstrated a similar pattern of enhancement after ultrasound processing[10]. This phenomenon may be explained by increase in bioactive substances, including bound polyphenols, flavonoids, and lycopene[26], as evidenced in our study. Previous study had also demonstrated that the increase in phenolic compounds was positively correlated with antioxidant activity during high-intensity ultrasound treatments[47]. Moreover, It had been proposed that another reason for enhanced antioxidant activity might be related to the endogenous enzymes inactivation by cavitation during thermal ultrasound process[48].

### 3.7 Electronic nasal analysis

**Fig. 5:**
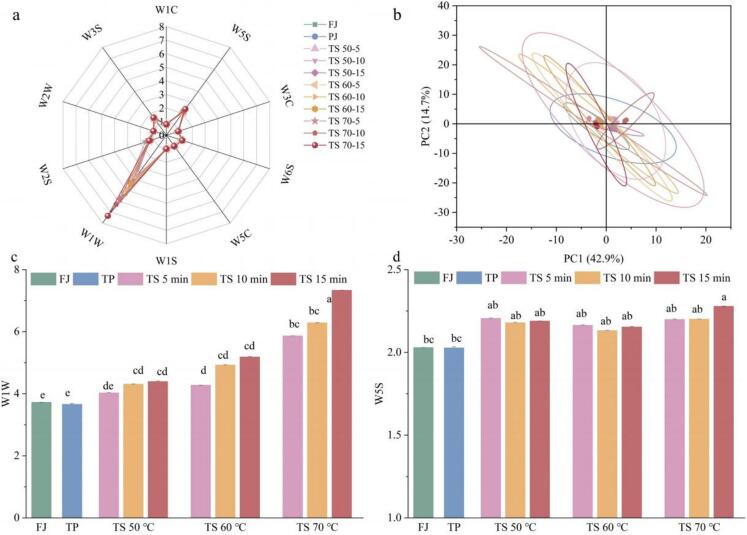
Effects of different sterilization methods on the volatile substances of tomato juices. (a) radar chart analysis of tomato juice; (b) principal component analysis (PCA) results of tomato juice; (c) sensor responses values of W1W; (d) sensor responses values of W5S. Different letters presented above the error line indicate significant differences between the samples (*p* ＜ 0.05).

Sensor types of E-nose responding to the odor of treated tomato juices were identical, but the response values exhibited notable difference (Fig. 5a). The W1W demonstrated the most intense response, with a range of 3.65–7.33, closely followed by W5S, with values between 2.03–2.28The W1W showed the strongest response of 3.65–7.33, indicating that the volatiles in tomato juice were predominantly organic sulfides and nitrogen oxides compounds. The response intensities of W1W and W5S for TS treated samples increased markedly in comparison to the FJ sample, suggesting that TS treatments exerted a considerable effect on the formation or release of flavor substances[49]. As shown in Fig. 5b, the points representing the FJ, PJ, and TS treated samples exhibited a high degree of spatial proximity, with occasional instances of overlap, suggesting that the TS treatments did not significantly change the overall odor distribution of the tomato juice. The initial two principal components collectively accounted for 67.6 % of the data variation (PC1 = 42.9 %, PC2 = 14.7 %), which could be regarded as significant components influencing the flavor of tomato juice. Results indicated that thermosonication did not modify the composition of the aroma components in tomato juice, but rather stimulated the content or concentration of these components. A similar conclusion has been reached in studies of melon juice[31] and black carrot juice[25].

### 3.8 Sensory evaluation

After TS treatments, all the sensory scores of tomato juice samples were superior to those of pasteurized samples as summarized in Table 2. The scores of the TS treated samples in color, aroma, taste, mouth feel, and overall acceptability were 8.93 ± 0.15 ∼ 9.17 ± 0.10, 9.07 ± 0.15 ∼ 9.27 ± 0.06, 8.45 ± 0.13 ∼ 8.83 ± 0.13, 8.43 ± 0.15 ∼ 8.83 ± 0.32, and 8.77 ± 0.15 ∼ 9.12 ± 0.20, respectively, while those of PJ samples were 9.02 ± 0.03, 9.00 ± 0.36, 8.32 ± 0.36, 7.87 ± 0.35, and 7.87 ± 0.40, respectively. There were only minor differences in sensory scores among TS treated samples. The decrease in sensory scores observed in pasteurized tomato juice samples might be related to the accumulation of sulfur-containing substances, which manifested the thermal-induced off-flavors[50]. The cooked off-flavor substances produced during the pasteurization might be the thermal degradation of carotenoid and phenols (ferulic acid)[51]. That could also explain why the sensory scores and bioactive ingredients content (TPC, TFC, AAC, and TLC) pasteurized at 85 ℃ were lower than that of other treatments. The sensory scores of the TS treated tomato juices were slightly higher than that of the PJ samples. It was hypothesized that thermosonication inactivated the browning enzyme and facilitated the release of flavor substances[52]. Therefore, the TS treatments could improve the color, flavor, and aroma of tomato juice.

**Table 2:** Effects of different sterilization methods on the sensory of tomato juice.

Treatments	Color	Aroma	Taste	Mouth feel	Total acceptability
FJ	9.08 ± 0.18^a^	9.27 ± 0.21^a^	8.62 ± 0.20^abc^	8.57 ± 0.31^a^	8.97 ± 0.21^a^
PJ	9.02 ± 0.03^a^	9.00 ± 0.36^a^	8.32 ± 0.36^a^	7.87 ± 0.35^b^	7.87 ± 0.40^b^
TS50-5	9.07 ± 0.28^a^	9.23 ± 0.21^a^	8.67 ± 0.21^ab^	8.67 ± 0.23^a^	9.12 ± 0.20^a^
TS50-10	8.97 ± 0.21^a^	9.20 ± 0.20^a^	8.73 ± 0.16^ab^	8.73 ± 0.21^a^	8.93 ± 0.15^a^
TS50-15	9.17 ± 0.08^a^	9.22 ± 0.10^a^	8.68 ± 0.08^ab^	8.63 ± 0.32^a^	8.98 ± 0.10^a^
TS60-5	8.93 ± 0.15^a^	9.13 ± 0.06^a^	8.73 ± 0.15^ab^	8.67 ± 0.21^a^	8.87 ± 0.35^a^
TS60-10	9.12 ± 0.20^a^	9.13 ± 0.12^a^	8.83 ± 0.13^a^	8.83 ± 0.32^a^	8.77 ± 0.15^a^
TS60-15	9.17 ± 0.10^a^	9.27 ± 0.06^a^	8.77 ± 0.15^ab^	8.83 ± 0.15^a^	8.93 ± 0.35^a^
TS70-5	8.97 ± 0.31^a^	9.27 ± 0.15^a^	8.75 ± 0.05^ab^	8.63 ± 0.25^a^	8.87 ± 0.15^a^
TS70-10	8.97 ± 0.28^a^	9.07 ± 0.15^a^	8.67 ± 0.15^ab^	8.67 ± 0.15^a^	8.82 ± 0.24^a^
TS70-15	9.02 ± 0.10^a^	9.08 ± 0.13^a^	8.45 ± 0.13^ab^	8.43 ± 0.15^a^	8.77 ± 0.15^a^

Note: Data were expressed as Means ± SD (n = 3). Values noted identical superscript letters in the same column indicated no statistically significant difference. The values that differ in superscript lettering presented significant (*p* < 0.05).

## 4 Conclusion

The study demonstrated that the TS treated tomato juice possesses a natural flavor, fresh color, and high nutritional value, and the optimal condition is 60 ℃ 15 min. The microorganisms (bacteria, yeast & mold, and *E. coli)* in the tomato juice were completely inactivated, and no significant difference were found in basis parameters including TA, TSS, and pH. And the retention rates in color and suspension stability of tomato juice were greatly enhanced. Notably, the heat-sensitive nutrients such as lycopene and ascorbic acid accumulated more, and the content of total phenols, flavonoids were also higher. Besides, the sensory quality and volatile substances were superior to pasteurized samples. This study indicates that TS is a more appropriate sterilization technology for processing tomato juice.

## CRediT authorship contribution statement

**Limei Li:** Writing – review & editing, Writing – original draft. **Hang Su:** Formal analysis. **Lingling Pang:** Writing – review & editing, Visualization. **Yanfang Pan:** Resources, Project administration. **Xihong Li:** Methodology, Conceptualization. **Qing Xu:** Project administration, Data curation. **Jitian Song:** Supervision, Conceptualization. **Liping Qiao:** Writing – review & editing, Visualization.

## Declaration of competing interest

The authors declare that they have no known competing financial interests or personal relationships that could have appeared to influence the work reported in this paper.

## Data Availability

Data will be made available on request.
